# Treatment of Chronic Inflammatory Demyelinating Polyneuropathy: From Molecular Bases to Practical Considerations

**DOI:** 10.1155/2014/201657

**Published:** 2014-01-14

**Authors:** Paolo Ripellino, Thomas Fleetwood, Roberto Cantello, Cristoforo Comi

**Affiliations:** ^1^Department of Neurology, A.O.U. Maggiore di Novara, Amedeo Avogadro University, Corso Mazzini 18, 28100 Novara, Italy; ^2^Department of Translational Medicine, Section of Neurology and Interdisciplinary Research Centre of Autoimmune Diseases (IRCAD), Via Solaroli 17, 28100 Novara, Italy; ^3^Department of Translational Medicine, Amedeo Avogadro University, Via Solaroli 17, 28100 Novara, Italy

## Abstract

Chronic inflammatory demyelinating polyneuropathy (CIDP) is an autoimmune disease of the peripheral nervous system, in which both cellular and humoral immune responses are involved. The disease is clinically heterogeneous with some patients displaying pure motor form and others also showing a variable degree of sensory dysfunction; disease evolution may also differ from patient to patient, since monophasic, progressive, and relapsing forms are reported. Underlying such clinical variability there is probably a broad spectrum of molecular dysfunctions that are and will be the target of therapeutic strategies. In this review we first explore the biological bases of current treatments and subsequently we focus on the practical management that must also take into account pharmacoeconomic issues.

## 1. Introduction

Chronic inflammatory demyelinating polyneuropathy (CIDP) is a peripheral nervous system disease that is clinically characterized by symmetrical, proximal, and distal weakness with altered sensation and hyporeflexia or areflexia [1]. Clinical course can be either relapsing remitting (RR), chronic progressive (CP), or monophasic [2]. In rare cases, CIDP displays acute onset and fast deterioration in the early phases, followed by chronic progression. This variant of CIDP, defined as “acute onset CIDP,” is difficult to distinguish from Guillain-Barré syndrome (GBS) in early disease stages [3]. Epidemiological studies on CIDP report an incidence in Northern Italy around 0.6 cases per 100.000 [4]. Nevertheless, it is probable that the real incidence of CIDP is largely underestimated, due to the variety of clinical presentations and the absence of proper diagnostic markers. For this reason, a diagnosis of CIPD must be taken into consideration while examining any polyneuropathy of unknown cause.

CIDP is an autoimmune disorder, as demonstrated by a great deal of evidence [5], such as the finding of inflammation at the site of the lesion [6], response to immunomodulatory treatment [7], and possibly the presence of autoantibodies against myelin antigens [8].

Long-term prognosis of CIDP has been correlated to age at onset, response to treatment, and time from onset to the beginning of treatment: young patients with acute onset are more likely to respond to treatment than elderly ones and proximal impairment has been linked to a better prognosis than distal weakness [9, 10]. The main negative prognostic factors of CIDP are progressive course and axonal degeneration [11].

CIDP and multiple sclerosis (MS) display similarities in clinical course and pathogenesis and there are reports on cooccurrence of these two demyelinating disorders [12], but no definite conclusion whether such event was coincidental or due to common mechanisms has been reached.

Peripheral nerve injury results from a synergistic interaction of cell-mediated and humoral immune responses directed against peripheral nerve antigens that have not been completely characterized [13].

From laboratory experiments we know that the key players in the pathogenesis of the disease appear to be T cells, especially T helper 1 (Th1) and T helper 17 (Th17) on one side and T regulatory (T reg) on the other [14]. A relevant contribution is also ascribed to the macrophagic component, cytokines, and complement activation [15–17].

CIDP is defined by a slow clinical deterioration that reaches its maximum after more than 8 weeks, differently from GBS, which is an acute and self-limiting disease. That aside, there are many similarities between these two conditions, which may even be variants of the same disease spectrum, with CIDP being the result of prolonged survival of activated T cells, not undergoing apoptosis due to a defective Fas pathway function [18–20], and GBS characterized by a self-limitation likely related to a preserved function of such apoptotic mechanism. In line with this concept, the finding that corticosteroids are effective in CIDP and not in GBS would be related to the known effect of these drugs in restoring T cell apoptosis.

Since inflammation is the core of the disease, it is not surprising that immunomodulatory treatments have a positive effect [21]. Nevertheless, it is not yet possible to predict disease progression on the basis of biological markers [22, 23] because it is likely that under the general definition of “CIDP” a broad spectrum of different forms is included [24].

In the following sections we will first discuss the biological basis for the use of immunomodulatory treatments in CIDP and subsequently illustrate our current strategy for choosing the best treatment option in everyday practice.

## 2. Biological Activity of Available Treatments

Currently available treatments for CIDP are corticosteroids, immune globulin, plasma exchange (PE), and chronic immunosuppressive agents [21, 25].

### 2.1. Steroids

Since the first report [26] of their use in CIDP in 1958, steroids have been considered a first-line therapy in CIDP. Nonetheless, their mechanism of action in patients with CIDP is not completely elucidated.

Many effects are mediated by intracellular receptors that modulate the expression of targeted genes [27]. The result of gene modulation is a pleiotropic anti-inflammatory effect mainly related to modulation of cytokines and to facilitation of apoptosis of T cells directed against the peripheral nerves [28, 29], as proved in animal models [30, 31] or in multiple sclerosis in humans [32]. During high-dose pulse therapies additional effects could occur, such as interference with intracellular signal transduction and interaction with activation of membrane-associated proteins.

A possible explanation for the variability in the clinical effect of glucocorticoids among patients and in the same patient, according to the stage of the disease, is alternative splicing. The alpha isoform of the glucocorticoid receptor (GR*α*) is a ligand-activated transcription factor. Alternative splicing of the glucocorticoid-receptor gene results in the expression of a GR*β* isoform that exhibits negative activity [33].

A “resistant state” to steroids—that is, a reduced response to glucocorticoids or the need to increase the dose—has been described in many autoimmune conditions [34] and seems to be induced by proinflammatory cytokines [35], increased GR*β* expression, or decreased glucocorticoid receptor binding. This state of glucocorticoid resistance could be positively influenced by concomitant treatment with intravenous immune globulin (IVIg) [36] with mechanisms that are still unclear but may include suppression of proinflammatory cytokines [37].

### 2.2. Intravenous Immune Globulin (IVIg)

The notion that the effect of intravenously administered immune globulin (IVIg) is not limited to antibody replacement is well established. Since the first demonstrations at the beginning of the 80s [38], it has become clear that IVIg plays a role in immunomodulation and has anti-inflammatory properties. However, the anti-inflammatory activity of IVIg is still to be understood and cannot be attributed to one specific mechanism of action but rather to a variety of different ones, acting at different levels and involving both innate and adaptive immune systems [39, 40]. Specifically, anti-inflammatory activities are seen when IVIg is administered at relatively high doses compared to those used for antibody replacement. On the contrary, low doses of IVIg seem to carry out an opposite proinflammatory activity possibly through the interaction with complement and activating receptors for the crystallizable fragment portion of IgG (Fc*γ*Rs) [39]. IVIg is a preparation of human polyclonal IgG obtained from plasma of several thousands of healthy donors [41], but it also contains traces of IgA and soluble molecules among which are cytokines, chemokines, soluble cytokine receptors, and receptor antagonists [39, 40]. Indeed the anti-inflammatory activity of IVIg can be related to the presence in the preparation of antibodies directed against serum proinflammatory molecules. However, IVIg seems also to act by modulating responsiveness to glucocorticoids, enhancing their anti-inflammatory effect [36].

IVIg contains antibodies with different specificities, but every antibody has the same structure: a variable portion called antigen-binding fragment (Fab) and a fixed fragment named crystallizable fragment (Fc) and both have been associated, in different ways, with anti-inflammatory activities [41, 42]. Some of the Fab-mediated activities may include neutralization of autoantibodies, cytokines, and activated complement components, anti-idiotype activity directed against autoreactive lymphocyte clones, modulation of cell migration, targeting of specific immune cell-surface receptors, and modulation of dendritic cells function. Fc-mediated activities instead may include blockade of the neonatal Fc receptor (FcRn) and activating of Fc*γ*R receptors on macrophages and other immune effector cells, upregulation of inhibitory receptor Fc*γ*RIIB, and immunomodulation by sialylated IgG [43–45].

A further role of IgG may be related to a reduction of complement uptake as they can bind to complement fragments such as C3, C4b, and C5 preventing tissue damage [46]. FcRn is found in many tissues and its activity increases the half-life of circulating IgG, as it normally binds to the Fc fragment and prevents IgG catabolism. High doses of IVIg may lead to saturation of FcRn with a consequent reduction of the half-life of autoantibodies [47]. However, this receptor displays particular affinity for deglycosylated IgG only and this aspect, which will be later discussed, tends to rule out this hypothesis [39]. Activating Fc*γ*R receptors also appear to be involved, as they play a key role in the triggering of effector functions in all myeloid cells. IgG in the preparation of immune globulin may bind to activating Fc*γ*Rs in the form of immune complexes thus blocking the interaction between autoantibodies and antigens. It has also been put into evidence that IgG2a and IgG2b subclasses display a greater capacity to initiate effector responses and this can be correlated to their higher affinity for activating Fc*γ*Rs [39, 48].

Moreover IVIg is thought to be able to induce an upregulation of inhibitory Fc*γ*RIIB receptor on effector cells whose function is to balance the activity of activating Fc*γ*Rs, dismissing inflammatory response by delivering inhibitory signals [48, 49]. This theory is supported by several studies, including one conducted on patients affected by CIDP [50]. Another important aspect of IgG function is the role of glycosylation in the interaction with Fc*γ*R receptors [51]. In detail, it seems that deglycosylated IgG fails to bind to such receptors [52]. Moreover, it appears that only a small percentage of glycosylated IVIg with *α*-2,6 sialic acid linkages on Fc-linked glycans is able to exert anti-inflammatory functions [53, 54] and this could explain why high doses of IVIg are needed to observe anti-inflammatory effects [47].

It has been suggested that sialylated Fc fragments may not directly interact with Fc*γ*Rs on effector cells, as they show reduced affinity, but that they may modulate inflammatory activity by binding to SIGN-R1 (ICAM-3 adhesion molecule) expressed on regulatory macrophages leading to the release of soluble mediators. These mediators would then bind to effector macrophages increasing the expression of inhibitory Fc*γ*RIIB which would eventually outcompete activating Fc*γ*Rs, increasing the number of immune complexes needed to trigger an inflammatory response [39, 54]. Immunomodulation by glycosylation leads to further considerations on the complex environmental regulation of immune responses and weakens those hypotheses based on simple IgG-Fc*γ*R and IgG-FcRn interactions. However many of these considerations have been derived from studies on animal models and must still be validated for humans. In spite of the fragmentary understanding of IVIg anti-inflammatory activity, immune globulin is successfully used in several autoimmune and inflammatory conditions including CIDP [55]. As already observed for steroids, response to treatment is often variable and this may be linked to genetic differences in immune system regulation [56] as well as glycosylation patterns in IVIg preparations.

### 2.3. Plasma Exchange (PE)

There are two main techniques of standard therapeutic plasmapheresis (or plasma exchange, PE): on-line plasma separation by a cell separator (centrifuge) or by a plasma separator (i.e., membrane filtration). A standard PE protocol for neuromuscular disorders employs 4 to 5 exchanges of 1 or 1.5 plasma volumes over one week or longer (until the patient shows satisfactory improvement) [57].

The aim of this treatment is the rapid removal of circulating autoantibodies, cytokines, immune complexes, and immune cells [58] and therefore PE is used in neuroimmunological antibody-mediated diseases [59] (e.g., myasthenia gravis) to achieve fast immunosuppression. However, the duration of these effects is limited in time because of resynthesis (or even rebound production) of the respective autoantibodies and therefore PE is combined with immunosuppressive medication in chronic diseases.

PE is traditionally used in acute forms of dysimmune peripheral neuropathies such as GBS, but also patients with chronic disease such as CIDP may respond to PE in the short term, usually for 2–4 weeks [60].

Although there is now no age limit for this treatment, several possible complications, such as cardiovascular systemic reactions, electrolyte disturbances, sepsis, thrombosis and thrombophlebitis, pulmonary embolism, and subacute bacterial endocarditis, limit its chronic use in elderly patients or in patients with multiorgan disease.

### 2.4. Immunosuppressive Drugs

Azathioprine (AZA) is an antimetabolite drug that interferes with the purine pathway and therefore with DNA synthesis in cell division; it causes inhibition of proliferating lymphocytes and is often used as steroid-sparing agent.

Methotrexate is another antimetabolite interfering with the synthesis of DNA and RNA and is commonly used in autoimmune diseases (e.g., rheumatoid arthritis).

Ciclosporin A inhibits the proliferation of T cells; its action seems to be much faster than the one of azathioprine.

Mycophenolate mofetil belongs to the antimetabolite group and is a prodrug; it inhibits the proliferation of T and B lymphocytes and is generally well tolerated and relatively safe to use, although side effects include mild bone marrow suppression.

Cyclophosphamide is an alkylating agent that can be given orally or by intravenous injection to deplete T and B lymphocytes. Neutropenic infections and transient renal insufficiency as well as other mild adverse effects have been reported, but the most common adverse effects are hemorrhagic cystitis, stomatitis, leukopenia, thrombocytopenia, malignancy, and cardiomyopathy. Therefore, cyclophosphamide should only be considered for patients with a severe form of CIDP who have been refractory to other treatments.

Rituximab is a chimeric (mouse/human) monoclonal antibody directed against CD20+ B lymphocytes. It is commonly used in lymphoma and has been tried on a small series of patients with paraproteinaemic demyelinating neuropathy, with modest benefit in selected patients.

## 3. Criteria of Treatment Choice in the Era of Pharmacoeconomy

Treatment choice will depend on several variables such as initial disease severity, age, general health status, and potential contraindications [61]. The recent economic crisis is opening remarkable questions about the sustainability of expensive drugs such as IVIg in Western countries and some national audits [62] or studies [63–65] have been already performed or are still ongoing (e.g., the prospective observational study “TEPORE” in Northern Italy) to clarify this issue. The treatment with immune globulin is highly expensive, especially for chronic patients, and there are concerns about future supplies because the pool of donors is decreasing and there is the need to improve microbiological screening of plasma donors.

Subcutaneous formulation of immune globulin is now available and offers an alternative to intravenous infusions, especially for patients in working age [66].

Patients with pure motor CIDP should be treated with IVIg, since deterioration has been reported with steroids, as in MMN [67].

If a patient has only mild symptoms, a nerve biopsy could help to confirm the diagnosis and establish the need for intervention if axonal degeneration has already occurred. Mild symptomatic patients should be followed up regularly with repeated neurophysiological examinations since relapses are unpredictable and oblige to start the treatment.

Some patients will not relapse after this first course, whereas some others (with relapsing-remitting form) will need additional treatment that should be individually tailored to achieve the most cost-effective regimen.

If a patient does not respond to one of the first-line therapies, switching to another is advisable.

PE or a combination of steroids and IVIg can be started if neither of these treatments proves effective. Refractory cases may need intensive immunosuppression, according to the general principle in medicine of escalating treatment for severe disease [57].

Long-term maintenance therapy will require careful attention because of side effects of treatments on the one hand and because of the risk of relapse and axonal loss on the other. Randomized clinical trials (RCT) with azathioprine, methotrexate, or other immunosuppressive agents could not provide evidence for their use as steroid or IVIg-sparing treatments, but none of these trials was large enough to rule out a small or moderate benefit.

### 3.1. Scores for Clinical Evaluation

CIDP diagnosis should be as accurate as possible in agreement with EFNS 2010 guidelines [1]. Such criteria are quite accurate and provide a better sensitivity compared to restrictive AAN criteria [68].

Patients with very mild symptoms, not or only slightly interfering with activities of daily living, may be monitored on a yearly basis by clinical examination, nerve conduction studies, and electromyography (EMG); in selected cases, when the diagnosis is not ascertained, sural nerve biopsy might be performed.

To evaluate disability progression, several scales have been proposed. In our opinion the Rankin Score, originally proposed for stroke patients and modified in 1988, lacks sensitivity to detect mild improvement occurring in the treatment of immune-mediated polyneuropathies [69]. Therefore, to monitor disability in the follow-up setting, the INCAT Overall Disability Sum Score (ODSS) should be preferred [70]. This score covers not only mobility disturbances but also upper limb dysfunction. Moreover, it has good clinimetric properties; it captures a high proportion of variance of disability and shows a good correlation with patients' perceptions [71, 72]. A recent report suggested that the Rasch-built Overall Disability Scale, a scale that specifically captures activity and social participation limitations in patients with autoimmune demyelinating polyneuropathies, might detect ability levels better than INCAT score [73]. Other authors suggest separating the screening for motor and sensory deficits when evaluating CIDP patients, as only the motor scores correlate with CIDP disease activity status (CDAS) [74]. The CDAS is a classification focused on the long-term evolution of CIDP [75].

For muscular strength evaluation, the Medical Research Council (MRC) sum score is historically used [76], even though a recent and large study conducted on patients with neuromuscular diseases underlined possible limitations of this score and proposed a simplified and probably more reliable version, referring to only four response categories [77].

On the other side, as a pure sensory score, the INCAT Sensory Sum Score (ISS) has been proposed more than ten years ago [78]. In our experience, distal sensory deficits, with or without neuropathic pain, often persist even in aggressively treated CIDP patients. This is probably due to irreversible axonal loss, but it rarely contributes to functional disability. The strong correlation between motor scores and the disability scales could be explained by the fact that disability is mainly due to the motor impairment: patients could refer to increasing tingling and numbness without a change in the INCAT score.

Finally, the small fibres damage cannot be measured with standard EMG techniques, but it could be quantified in clinical trials or research setting with quantitative sensory testing (QST) and laser evoked potentials.

### 3.2. Newly Diagnosed Patients: First-Line Treatments

Treatment with corticosteroids or IVIg should be offered to patients with moderate or severe disability [1].

The efficacy of steroids in CIDP in the short term has been repeatedly proved, first compared to placebo [79, 80] and then to IVIg: in 2001 a controlled study has shown that a 6-week course of 60 mg daily oral prednisolone with rapid tapering is as effective as one course of IVIg at 2 g/kg [81].

If there is no major contraindication (such as diabetes or prediabetic stages as impaired fasting glucose or impaired glucose tolerance) and since it is not possible to predict if the patient will be steroid responder or not, we prefer to try steroids first because of the need of a spending review [82] and because long-term remission can be achieved in about one-quarter of patients with CIDP after 1 or 2 courses of pulsed dexamethasone or 8-month daily prednisolone [83]. Intravenous or oral methylprednisolone [84], oral prednisolone [81], and intravenous dexamethasone [85] are all validated treatments.

However, as in other autoimmune disorders, long-term steroids as monotherapy are usually not recommended because of side effects (Cushing's syndrome, cataracts, glaucoma, diabetes, hypertension, weight gain, osteonecrosis, gastrointestinal ulcer, psychiatric disturbances, peripheral edema, hypokalemia, myopathy, and increased risk of infections).

According to literature, there is no consensus about whether to use daily or alternate-day prednisolone or prednisone or intermittent high-dose monthly intravenous or oral regimens. The generally accepted dosage for prednisolone is 60 mg/day (1-1.5 mg/kg) as induction therapy up to 12 weeks; if there is a response, the dose should be tapered to a low maintenance level over 1 or 2 years and eventually corticosteroids can be withdrawn [1]. Both daily dosing and alternate-day dosing for the oral treatment have been employed [86]. However, to our knowledge, if corticosteroids are chosen as first-line treatment intravenous pulsed therapy seems to be a more appropriate choice: the PREDICT study [85] could not show a significant difference in terms of duration of remission between pulsed high-dose dexamethasone and oral prednisolone for 6 months, but the intravenous treatment led to a faster improvement, relatively fewer relapses, and less adverse events.

Our favourite first-line therapy is methylprednisolone 500 mg IV for 4 consecutive days in the morning, every month for 6 months, but the efficacy in the short term should be equivalent to dexamethasone and prednisolone. Once IV pulsed steroid treatment shows clear cut improvement (clinical and on nerve conduction studies), an immunosuppressive agent, such as azathioprine, can be introduced in addition to oral maintenance therapy with prednisolone or prednisone at a dose of 60–80 mg/day until major improvement is seen.

Subsequently, oral steroids can be tapered, but if the patient in remission experiences a relapse, one may consider repeating the course of corticosteroids, especially if the first course led to a long-term sustained remission.

As second-line treatments two opposite approaches could be used and have equivalent effects: PE and IVIg [60].

If a patient does not respond to one of these first-line therapies, it is advisable to switch to the other one [87], but it is never clinically advisable to perform PE few days after an IVIg course.

PE leads to rapid improvement in disability, clinical impairment, and motor nerve conduction velocity in CIDP [88]; however, the main limitations of PE are the short-term benefit (usually 2 weeks) and the rate of side effects related to difficulty with venous access, use of citrate, and haemodynamic changes [89].

Repeated treatments are usually required. The optimal number of plasma exchange treatments has been reported for the acute form GBS: in one large multicenter study [90] it was shown that 2 PE are optimal for mild GBS, whereas 4 PE should be reserved for patients with moderate/severe forms.

Polyclonal human immune globulin infusion is a highly effective treatment based on multiple and still unknown mechanisms of action [43], but its cost is comparable or lower than that of PE [91] and it has fewer side effects compared to PE. The maximal clinical response to IVIg should be evident after 2 weeks from the infusion [92]. The therapeutic benefit of IVIg in CIDP in the short term was evaluated by few RCT in the 90s [93–95]. The benefit was greater for acutely relapsing patients and was reproducible after subsequent infusions [96].

In 2001 another RCT focused on 30 naïve patients and showed that IVIg is also effective as initial treatment [97].

Plasma exchange and IVIg are equally efficacious in the short term in CIDP patients [98], but PE is much less practical for maintenance treatments.

The short term efficacy of IVIg compared to placebo is supported by a large clinical trial on 117 CIDP patients called the ICE study. More than 50% of patients treated with IVIg had an improvement in the INCAT score, compared to only 20% of placebo-treated patients [55]. After this trial the use of IVIg spread over and neurologists had to consider the option of IVIg as a starting treatment [99].

The efficacy of IVIg in CIDP has been confirmed by a Cochrane review: IVIg improves disability for at least two to six weeks compared with placebo, with a number needed to treat of 3 and efficacy similar to PE or oral prednisolone [100].

In our opinion IVIg is a first-line treatment in CIDP patients with a proven contraindication to steroids and a second-line treatment or add-on treatment in patients who do not reach the expected improvement with steroids. We also prefer IVIg treatment from the beginning in patients with pure motor CIDP for whom a possible clinical worsening under steroids has been reported in a small patients series [67].

The switch from steroids to IVIg has to be considered if the patient is developing severe side effects (diabetes, osteopenia or osteonecrosis, Cushing, or cataract), if the patient is pregnant or wants to become pregnant, or if the patient is worsening more quickly than expected.

The standard dose for starting IVIg is 0.4 g/kg for 5 days (totally 2 g/kg) after checking IgA serum concentrations. The first dose is fractionated to reduce the risk of possible side effects or intolerance. Some side effects are correlated with the speed of administration; the patient should be monitored for headache, sweating, thoracic discomfort, and the dose administered over a longer time of infusion (4-5 hours).

The maximal clinical response to IVIg should be evident after 3 weeks from infusion [92]. It is advisable to schedule a follow-up visit in an outpatient setting after 1 month and after 2 months from the beginning of treatment. The second infusion of IVIg could be administered after an interval of 6 weeks from the previous infusion and during the second consult the neurologist should establish a schedule for maintenance infusions. Our standard maintenance dose is 0.5 g/day for 2 consecutive days (1 g/kg/month); a different dose should be carefully evaluated by the neurologist, according to the principle of the “lowest effective maintenance dose” [101]. Follow-up visits on a regular basis (every 4–6 months) may help to decide if the IVIg dose should be modified.

Combined treatment of steroids and IVIg from the beginning is used in other severe autoimmune diseases (e.g., myasthenia gravis), but this has to be considered an *off-label *strategy.

For patients with acute onset CIDP or with a severe form of CIDP from the beginning (INCAT score of 3 or more) we prefer treatment with IVIg or plasma exchange (with 5-6 exchanges of 1-1.5 plasma volumes over 10 days) plus steroids and an immunosuppressive agent until major improvement is noted.

### 3.3. Patients Already in Follow-Up

We always reconsider the diagnosis of CIDP if the patient does not respond to first-line treatment and in any case before starting an immunosuppressive drug, especially when there could be an underlying paraproteinemic polyneuropathy. In case of disease progression despite treatment, sural biopsy [17, 102] and repeated EMG studies could help to differentiate CIDP from other polyneuropathies from other forms.

The combination of the INCAT score plus neurophysiological follow-up could help to objectify the clinical improvement. A definition of “responder” based on disability has been suggested by Cocito and coauthors in a retrospective observational analysis [25]: responders were those patients who had an improvement of at least one point in the Rankin Scale after therapy. About 70% of patients responded to first-line immunological therapies: 61% to steroids, 73% to IVIg. Lack of response to one treatment did not preclude a response to another treatment: about 50% of the nonresponders to first-line therapy became responders when switched to an alternative drug, so that after a single switch the percentage of responders reached 80% and this proportion could be higher if treatments are combined.

Long-term treatment with corticosteroids has proved to be effective [103] but is hampered by the development of side effects that often cannot be adequately captured in short-term trials, even those with one-year follow-up. To reduce side effects, regimens alternatives to the standard 12 weeks oral prednisolone followed by 1-2 years with slow tapering have been suggested in the PREDICT study [85].

Recently, the long-term follow-up of the PREDICT study [83] provided evidence that 1-2 courses of pulsed IV dexamethasone or 8-month daily oral prednisolone allowed cure or remission in 25% of 39 CIDP patients followedup for more than 4 years.

During the monitoring period, the clinician should prevent steroid-related side effects: every patient should be provided with calcium, vitamin D, and proton-pump inhibitors. A careful monitoring of blood pressure, weight, blood sugar, and osteopenia is mandatory in all patients treated with steroids for more than 3 months.

The efficacy of long-term treatment with IVIg has been investigated in retrospective studies in comparison to PE [104] or to other treatment options in smaller [105] or larger series [25].

11 CIDP patients treated for one year with IVIg were evaluated in a neurophysiological study that showed a decrease in the rate of conduction blocks and axonal loss [106]. The most reliable and consistent data about long-term efficacy and safety of IVIg treatment come from the extension phase of the ICE study [55]: relapse rate was significantly lower in IVIg-treated patients compared to patients who received placebo, with a side effects rate comparable between the two arms. Periodic IVIg administration significantly sustained the initial improvement seen in CIDP patients and this effect could last months without reinfusions in a significant proportion of patients. In fact, 55% of patients rerandomized to placebo did not relapse after 24 more weeks.

A recent retrospective study [107] focused on the long-term effect of IVIg in 87 Spanish patients evaluated after more than 48 weeks, with or without concomitant immunosuppressive medication. The dose of IVIg was individualized for each patient, whereas doses and frequencies were fixed in the ICE trial. The main finding of this study is that about one-quarter of patients were stable at least 6 months after the last IVIg infusion, suggesting that in the long term a careful reevaluation of the patient conditions is mandatory to avoid overtreatment and reduce costs for the healthcare system: the optimal frequency and dose of IVIg infusion should be individualized according to the patient's need and disease course, as also stated in the EFNS guidelines [1].

Since the costs of this therapy are very high, the neurologist should find the lowest effective maintenance dose. In patients treated for years a temporary withdrawal could also be attempted: this observation time could help to decide if the patient has still, after years, a real benefit from the treatment, because patients may need less IVIg than they receive or in fact none at all. In an international study the IVIg dose could be reduced by over 20% without deterioration in almost half of the patients [108].

Some preliminary reports suggest that subcutaneous immunoglobulin may be as effective as IVIg in the maintenance therapy of CIDP [109, 110].

In 2012 IVIg and IV methylprednisolone (MP) treatments were directly compared in a multicenter, randomized, double-blind, placebo-controlled, parallel-group study [111]. This study provided evidence that the efficacy of MP is comparable to IVIg, but there are some differences concerning tolerability and effect duration. A chronic treatment with steroids is associated with a higher rate of side effects but also with longer neurological stability. Overall, almost 50% of patient from both groups did not require any further infusion after one year since they showed either improvement or symptoms stability.

Nonetheless, it should be noted that those results cannot be translated to drug naïve patients, since this study included previously treated patients.

To sustain long-term remissions there is a need for “IVIg-sparing” agents [112], as IVIg infusions are required every 3–6 weeks. In CIDP, immunosuppressive drugs such as azathioprine, cyclosporine, methotrexate, mycophenolate, and cyclophosphamide are generally used [113], but a Cochrane meta-analysis concluded that there is no evidence that they are effective [114].

Many years ago an open-label, randomized, controlled trial [115] of 27 patients, comparing azathioprine in combination with prednisone to prednisone, alone showed no significant difference between treatments, although it should be noted that the sample size was small, the patient sample was extremely heterogeneous, and the treatment period of 9 months was too short to draw conclusions about efficacy, since several months have to pass before azathioprine reaches maximal effect.

Despite this RCT, azathioprine has been widely used in open label after initial PE or IVIg and corticosteroid treatment to maintain remission.

In patients with a long history of disease or in patients refractory to other treatments we start azathioprine at the standard dose of 2-3 mg/kg/day. This option seems to be desirable also for patients preferring a home therapy instead of a periodic access to the Day Hospital.

Data from a retrospective Italian study [116] suggest that about 25% of patients refractory to first-line treatment do respond to immunosuppressive agents (usually azathioprine or methotrexate for mild forms, cyclophosphamide for severe forms).

Oral methotrexate as a monotherapy in patients with CIDP has been compared to placebo in an RCT [108] in 60 CIDP patients who had previously responded to and were still receiving corticosteroids or IVIg. With a dose of 15 mg per week authors could not detect significant benefits, but limitations in the trial design and the high rate of responses in the placebo group meant that a treatment effect could not be excluded.

The larger study regarding cyclosporin A efficacy is based on a retrospective analysis of 19 Australian patients resistant to other therapies [117]; the efficacy of this drug is counterbalanced by kidney failure, an important side-effect.

There are few reports on the use of mycophenolate mofetil in CIDP in small series of patients with conflicting results [118–120].

Two small series of 15 [121] and 5 patients [122] treated with IV pulsed cyclophosphamide gave beneficial results, but the toxicity of this drug limits its use to refractory cases.

According to uncontrolled studies [123–125] Rituximab might be helpful in CIDP associated with hematological disorders such as monoclonal gammopathy of undetermined significance and multiple myeloma, but a very recent RCT [126] on 54 patients with anti-MAG followed up for one year has shown no significant benefit from Rituximab compared to placebo in terms of changes in the ISS score [78]. Rituximab also failed as an IVIg-sparing agent in patients dependent on IVIg [127].

Retrospective analysis based on large population studies shows that a certain proportion of CIDP patients remain free of disease in the long term, regardless of their treatment regimens; whether disease activity cessation was due to treatment effect or to spontaneous remission of the disease remains unknown [75, 107].

## 4. Conclusions

CIDP is a rare but treatable disease. Clinical experience indicates that about 70% of patients will respond to immunomodulation; there are patients responding to steroids, whereas others, especially with pure motor CIDP, will benefit more from IVIg or PE. It is becoming evident that CIDP is not a uniform disease but includes several variants which might have different response profiles.

First-line treatment choice depends on several factors, such as disease severity, the presence of a pure motor form of CIDP, contraindications and side effects of long-term therapy, costs, and local availability of PE or IVIg.

We would encourage international guidelines specifically devoted to define an algorithm for first-line therapy and for standardized follow-up.

There is a need to improve the identification of CIDP subforms not only in terms of clinical presentation (typical versus atypical) but also therapeutic response [75], especially for the IVIg treatment. It is currently under debate whether the profile of IVIg is favourable enough to outweigh the higher costs associated with its long-term use.

In conclusion, as it is mandatory to avoid overtreatment in benign forms, it is crucial to achieve long-term remission in severe forms. A short-term intensive treatment may help prevent prolonged use of corticosteroids or IVIg. In our experience, immunosuppressive agents are helpful in this long-term strategy even if, until further controlled clinical trials are available, they will remain off-label strategies.
